# Health Risk Assessment for Cyanobacterial Toxins in Seafood

**DOI:** 10.3390/ijerph9030807

**Published:** 2012-03-07

**Authors:** Vanora Mulvenna, Katie Dale, Brian Priestly, Utz Mueller, Andrew Humpage, Glen Shaw, Graeme Allinson, Ian Falconer

**Affiliations:** 1 Department of Health, GPO Box 4541, Melbourne, Victoria 3000, Australia; Email: vanora.mulvenna@health.vic.gov.au; 2 Department of Epidemiology & Preventive Medicine, School of Public Health and Preventive Medicine, Monash University, Melbourne, Victoria 3004, Australia; Email: katie.dale@monash.edu; 3 Australian Centre for Human Health Risk Assessment, School of Public Health & Preventive Medicine, Monash University, Melbourne, Victoria 3004, Australia; Email: brian.priestly@monash.edu; 4 Food Standards Australia New Zealand, 55 Blackall Street, Barton, ACT 2600, Australia; Email: utz.mueller@foodstandards.gov.au; 5 Australian Water Quality Centre, SA Water, GPO Box 1751, Adelaide, SA 5001, Australia; Email: Andrew.Humpage@sawater.com.au; 6 School of Public Health, Griffith University, Gold Coast Campus, Queensland 4222, Australia; Email: g.shaw@griffith.edu.au; 7 Future Farming Systems Research Division, Department of Primary Industries, Queenscliff, Victoria 3225, Australia; Email: graeme.allinson@dpi.vic.gov.au; 8 Pharmacology, Medical Sciences, University of Adelaide, Adelaide, SA 5005, Australia

**Keywords:** cyanobacteria, blue-green algae, toxins, seafood safety, health guidelines

## Abstract

Cyanobacteria (blue-green algae) are abundant in fresh, brackish and marine waters worldwide. When toxins produced by cyanobacteria are present in the aquatic environment, seafood harvested from these waters may present a health hazard to consumers. Toxicity hazards from seafood have been internationally recognised when the source is from marine algae (dinoflagellates and diatoms), but to date few risk assessments for cyanobacterial toxins in seafood have been presented. This paper estimates risk from seafood contaminated by cyanobacterial toxins, and provides guidelines for safe human consumption.

## 1. Introduction

The Gippsland Lakes are a system of coastal lagoons situated in southeast of Victoria (Australia), approximately 200 km east of Melbourne and are important for recreational, tourist and commercial activities. The Lakes are a commercial seafood source, including shellfish, crustaceans and fish, as well as providing popular recreational fisheries. Considerable modifications to the Lakes catchments have occurred since European settlement with agricultural and fisheries development, including the creation of a permanently open entrance to Bass Straight in 1889. This environment, which was once a freshwater lake, is now a more saline, high nutrient region, and cyanobacterial (blue-green algal) blooms have now become a common occurrence. Since 1985, there have been seven non-cyanobacterial blooms recorded in the Lakes (usually diatoms or dinoflagellates), and 12 cyanobacterial blooms [1]. *Nodularia spumigena* is the most frequent cyanobacterium to bloom, with sporadic *Anabaena* *circinalis* and *Microcystis aeruginosa* blooms.

Cyanobacterial blooms are largely dependent on nutrient availability and water temperature, and the predominant species affected by salinity. Warmer waters during summer accelerate growth of the organisms [2]. Cyanobacterial blooms are a public health concern because the toxins that some species of cyanobacteria produce can have harmful effects on consumers, whether this is through drinking water, recreational exposure or from seafood. All three common bloom-forming cyanobacteria in the Gippsland lakes are toxic species. *Nodularia spumigena*, which is abundant in the Lakes, was the first cyanobacterium to be identified in the scientific literature as the cause of livestock poisoning in 1878 [3]. Cyanobacterial toxins have been shown to bioaccumulate in aquatic organisms such as shellfish, prawns and fish, and have previously resulted in restrictions on the collection of these organisms from the Gippsland Lakes [4,5]. In many cases the toxicity is sublethal to these aquatic species, which allows the animals to survive long enough to accumulate toxins and transfer them along the food chain [4]. It is possible that the concentration of cyanobacterial toxins in seafood can reach levels at which human consumption should be discouraged [6]. With the exception of the worldwide adoption of guideline levels for saxitoxins in seafood (for example based on Food Standards Australia New Zealand (FSANZ) Food Standard 1.4.1), there are no national guidelines that advise on safe levels of cyanobacterial toxins in seafood.

In order to provide advice and to define acceptable levels of cyanobacterial toxins in seafood in Victoria, Australia, the Victorian Department of Health convened a scientific advisory group to carry out a risk assessment regarding commercial and recreational seafood safety in the Gippsland Lakes. The seafoods of concern were fish, prawns and mussels harvested from the lakes. The identified toxins for the risk assessment were microcystins, nodularin, saxitoxins and cylindrospermopsin, all of which have been found in Australian aquatic environments and are distributed worldwide [6].

## 2. Methods, Results and Discussion

### 2.1. Risk Assessment Methodology

The standard human health risk assessment approach was employed, which incorporates the following steps:

(1) Hazard identification—qualitative determination of the potential of a chemical or agent to cause adverse effects in humans.
(2) Dose-response assessment—examination of the quantitative relationship between the hazard at different exposure levels and the incidence of adverse effects in humans or other animals.
(3) Exposure assessment—determination of the route, frequency and duration of the exposure, including the nature of exposed populations.
(4) Risk characterisation—integration of hazard presence, dose-response and exposure assessment information.

### 2.2. Hazard Identification

In carrying out the hazard identification and dose-response assessment of cyanobacterial toxins in seafood, the majority of relevant studies identified had been undertaken on saxitoxins of marine dinoflagellate origin, with few reported cases of human cyanobacterial toxicity associated with seafood consumption [7]. In addition, none of the reported cases detailed the adverse effects in humans as a function of dose. However human toxicity from cyanobacterial toxin contamination of dialysis fluid, drinking water and recreational water consumption has been demonstrated. 

### 2.3. Dose-Response Assessment

With no direct information regarding the human health risks of cyanobacterial toxins in seafood as a function of degree of exposure (dose-response), it is necessary to extrapolate information from animal toxicity studies. There are several different types of animal studies used to identify hazards and to assess the dose-response. They include acute, sub-chronic, chronic, reproductive and developmental toxicity, as well as genotoxicity studies [8]. As only oral exposure is relevant when considering toxin exposure from seafood, animal studies in the published literature that were conducted in accordance with the OECD 1998 Guideline for the Testing of Chemicals for sub-chronic oral toxicity were assessed [8,9]. Where possible, experimental data from two species, one rodent and one non-rodent, was used.

To determine the safe dose of potentially toxic materials in the diet, toxicological data is used to calculate a Tolerable Daily Intake (TDI). This is an estimate of the intake of a substance which is without appreciable health risk to consumers over their lifetime. However, as the cyanobacterial toxins are acutely toxic and consumers may eat large portion sizes on occasions, it is also appropriate to consider establishing an acute reference dose (ARfD). The ARfD is an estimate of “the amount a substance in food or drinking water, normally expressed on a body weight basis that can be ingested in a period of 24 hours or less without appreciable health risks to the consumer on the basis of all known facts at the time of the evaluation” [10]. The ARfD is used to assess the dietary risk for those consumers who eat high levels of seafood in a single meal or over a single day, while the TDI is used to assess the dietary risk for those consumers that eat the average level of seafood over a lifetime. Both the TDI and ARfD are calculated from experimental data for the intake of the substance which has no detectable adverse health effects, called the No Observed Adverse Effect Level (NOAEL).

Uncertainty factors are applied to the NOAEL to allow for variations in individual sensitivity, extrapolation between human and animal studies and to account for uncertainties in data. The standard factors are 10 for intraspecies (within human) variability, 10 for interspecies (rodent compared to human) variability, and an additional (third) variable factor for limitations in data. Limitations in the data that this additional factor may account for include: the use of only one sex or species of animal; possibility of mutagenicity or carcinogenicity; teratogenicity or reproductive toxicity. In the derivation of all health guideline values for cyanobacterial toxins in seafood (refer to Section 3 below) this additional factor was assigned a value of 2. For each of the toxins the limitations in the data that necessitated this value of 2 varied (see below). It should be noted, however, that for none of the toxins did this value aim to allow for the fact the toxicity trials were sub-chronic rather than lifetime. For these toxins this was not regarded as a limitation in the data because the seasonality and irregularity of toxic cyanobacterial blooms mean that human exposure to the toxins will be acute to sub-chronic. For each of the toxins the total uncertainty factor used in the derivation of the health guideline values was 200 (10 × 10 × 2)

### 2.4. Exposure Assessment

To convert the TDI or ARfD into a health guideline value in seafood it is necessary to undertake an exposure assessment to incorporate information including the bodyweight of consumers; the quantity of seafood consumed; and whether the consumer may be exposed to the toxin via other sources (for example, through drinking water or through recreational activities). Average body weights for specific age groups (that is 17 years or above and 2–16 years) were sourced from recent national nutritional surveys [11,12]. For acute dietary risk assessment purposes and to protect consumers of high levels of seafood, data on the high-level consumption of fin fish, prawns and molluscs (97.5th percentile intake) were extracted from the 1995 National Nutrition Survey (NNS) 1 for consumers aged 17 years and above (Table 1). For children aged 2–16 years, the more recent 2007 Australian National Children’s Nutrition and Physical Activity Survey 2 was used (Table 1).

It is not possible to establish generic health guideline values which would be applicable to other regions of the world because seafood consumption patterns can vary considerably. However, if average bodyweights and high-level consumption data are available then it is possible to use the outlined procedure to derive relevant health guideline values for other countries where seafood consumption patterns differ substantially from Australia and New Zealand.

**Table 1:** High level seafood consumption (97.5th percentile).

Age group (years)	Commodity	Consumer intake ^4^ g/kg BW ^5^/day	g/day	Survey ^1,2^
≥17	Fish (diadromous and freshwater) ^3^	5.1	377	1995
	Prawns	5.1	377	
	Mussels	2.4	178	
2–16	Fish (diadromous and freshwater	8.4	319	2007
	Prawns	6.2	236	
	Molluscs ^6^	3.9	148	

Notes: ^1^ 1995 National Nutrition Survey of Australia (1995 NNS), surveying 13858 people aged 2 years and over. This survey used one 24-hour recall for all respondents and a second 24-hour recall for approximately 10% of respondents [11]; ^2^ 2007 Australian National Children’s Nutrition and Physical Activity Survey (2007 NCS), surveying 4487 people aged 2–16 years. The survey used two 24-hr recalls for all respondents. For the purposes of estimating acute dietary exposures only a single day 24-hour recall is used [12]; ^3^ Diadromous and freshwater fish consumption excluding all marine fish; ^4^ The food consumption data outlined in the table includes the amount consumed alone and as an ingredient in mixed foods. In DIAMOND, FSANZ’s dietary exposure assessment computer program, all mixed foods have a recipe and these recipes were used to break down mixed foods into their raw commodity components. For example fish contained in a fish casserole are included in fish consumption; ^5^ Bodyweights: 74 kg > 17 years; 38 kg 2–16 years; ^6^ Consumption amounts for molluscs were used where there were insufficient consumers of mussels alone to derive a valid 97.5th percentile consumption value. There were only 11 consumers of mussels in the 2–16 year age group dataset. “Molluscs” includes mussels, octopus, oysters, scallops and squid.

### 2.5. Allocation Factor

In the context of this report the allocation factor is defined as the proportion of toxin exposure gained through the consumption of seafood. The only likely route of public exposure to cyanobacterial toxins would be through the consumption of seafood. Drinking water is not sourced from the Gippsland Lakes and recreational use of the lakes is strongly advised against as soon as a significant cyanobacterial bloom occurs, as per the *Guidelines for Managing Risks in Recreational Water* [13]. An allocation factor of one (or 100% of toxin exposure gained through consumption of seafood) was therefore deemed appropriate.

### 2.6. Risk Characterisation

It is appropriate to consider the acute dietary risks posed by the presence of cyanobacterial toxins in seafood as they are acutely toxic and consumers may eat large portion sizes on occasions. Therefore, the establishment of maximum levels (MLs) for seafood should ideally be based on an acute dietary risk characterisation, which would be suitably protective of excessive chronic exposures as the target organ is the same following either a single or repeated dietary exposure.

There is a paucity of data on the thresholds of acute oral toxicity for cyanobacterial toxins and therefore limited potential to establish an ARfD for cylindrospermopsin and microcystins. On this basis, a conservative approach has been taken where the high-level intake (the 97.5th percentile intake) of fin fish, prawns or mussels or molluscs have been compared with the TDI. Two population groups have been assessed; 17 years and above and 2–16 year olds. For the saxitoxins (STXs), no acute dietary risk characterisation has been undertaken because the current Australian and New Zealand ML has proven to be an effective risk management limit.

By integrating the hazard identification, dose-response and exposure assessment information, health guideline values for cylindrospermopsin and microcystins in seafood can be derived. The steps are as follows: 

Step 1: Determine the TDI (μg/kg bw/day):

*TDI (μg/kg bw/day) = No Observable Adverse Effect Level* *÷ Uncertainty Factors* (1)

Step 2: Determine the acceptable limit of toxin consumption per person per day (μg/day):

*Acceptable limit (μg/day) = TDI (or ARfD) × average bodyweight (kg) × allocation factor* (2)

Step 3: Define the high level intake of seafood per consumer per day (kg/day).

Step 4: Derive health guideline level for toxin in seafood:

*Guideline value (μg/kg) = Acceptable limit (μg/day)* *÷ consumption of seafood per day (kg/day)* (3)

Health guideline values need to be derived for each cyanobacterial toxin separately (using specific TDI or ARfD values) and then for each seafood (fish, prawns and mussels or molluscs) separately (using different average consumption values). 

## 3. Derivation of Health Guideline Values

### 3.1. Cylindrospermopsin

Cylindrospermopsin (CYN) occurs in fresh and brackish waters worldwide, due to the presence of the cyanobacterial genera *Cylindrospermopsis*, *Aphanizomenon*, *Anabaena*, *Raphidiopis*, *Lyngbya* and *Umezakia* [14].

Studies of bioaccumulation of cylindrospermopsin in gastropod snails, bivalves [15], crustaceans [16], amphibian tadpoles and fish [17] demonstrated that this toxin is concentrated into tissues from free solution and from toxic *Cylindrospermopsis* cells. The highest accumulation was seen in mussels with a whole-body concentration of almost 3 mg/kg dry weight, with the maximum tissue concentration found in haemolymph. Cylindrospermopsin appears in muscle tissue as well as viscera, increasing the possibility of consumption in these seafoods.

Human poisoning from CYN has been previously recorded. In Palm Island in 1979, for example, 150 people received hospital treatment for an unusual hepatoenteritis after drinking water from a reservoir that was treated with copper to remove a *Cylindrospermopsis* algal bloom [18]. The absence of toxin exposure information, however, makes this case unusable for the purposes of deriving a TDI. There have, however, been several published accounts of the oral toxicity of cylindrospermopsin in animals, with the majority of studies using a single dose [19,20,21]. Repeat oral dosing after a two week interval showed unexpectedly enhanced toxicity, indicating residual damage to the animals from the first dose [22].

A study by Humpage and Falconer [23], following the protocols set out by the OECD for subchronic oral toxicity assessment in rodents, exposed male Swiss Albino mice to cylindrospermopsin through drinking water and through gavage (dosing by mouth) [9]. The first trial used a cylindrospermopsin-containing extract from cultured *Cylindrospermopsis raciborskii*, supplied in drinking water for 10 weeks. The dose of cylindrospermopsin ranged from 0 to 657 μg/kg/day at 4 levels. The animals were examined clinically during the trial and showed no ill effects other than a small dose-related decrease in body weight compared to controls after 10 weeks. Liver and kidney weights were significantly higher with increasing dose. Several biochemical indicators of liver function showed dose-related changes. For instance, serum total bilirubin and albumin increased, while serum bile acids decreased. Liver enzyme changes in the serum showed a different pattern to those seen with acute liver poisoning or hepatitis, as only a small increase in serum alanine aminotransferase and a larger increase in alkaline phosphatase were observed. There was also a decrease in aspartate aminotransferase. The most substantial change observed was in the urine protein/creatinine concentration, which decreased sharply with dose. This was interpreted as reflecting decreased protein synthesis in the kidney through inhibition by the toxin. Histopathological examination of all internal organs showed changes only in the liver and kidney. Dose-related hepatocyte damage and renal proximal tubule necrosis were observed [23].

When it was apparent from these results that lower oral doses were required to find the No Observed Adverse Effect Level, Humpage and Falconer [23] carried out a second trial in which mice were dosed orally by gavage over 11 weeks with 0, 30, 60, 120 and 240 μg/kg/day of purified cylindrospermopsin. The same trends in serum parameters were seen, but with no statistically significant changes. Organ weights showed most sensitivity to these low doses with significant increases in body weight, and as a percentage of body weight, in liver, kidney, adrenal glands and testis. Minor histopathological damage was seen in the liver at the two upper dose levels, and in kidney proximal tubules at the highest dose. Urine protein/creatinine decreased progressively with dose, reaching significance at 120 μg/kg/day of oral cylindrospermopsin.

At very low dose levels of toxins compensatory changes occur in metabolism to restore homeostasis. The increases in organ weight can be expected to compensate for reductions in function as seen in the liver and kidneys, and compensation for stresses resulting from the toxin, for example in the adrenal glands. It therefore becomes subjective to decide where the No Observed Adverse Effect Level occurs, depending on which effect is considered adverse. From the urine protein data it is clear that the NOAEL is below 120 μg/kg/day. However statistically significant change in kidney weight occurred at 60 μg/kg/day.Thus to adopt the conservative viewpoint that the most sensitive response should be considered as the indicator of adverse effect, the dose of 30 μg/kg/day was accepted as the NOAEL from these trials [23]. Recent studies have corroborated this value and shown that both males and females are affected to a similar degree [24].

Thus using 30 μg/kg/day as the NOAEL, the TDI for cylindrospermopsin can be calculated.

*TDI(μg/kg/day) = 30* *÷ Uncertainty factors* (4)

The uncertainty factors are 10 for intraspecies variability, 10 for interspecies variability and an additional factor of 2 given that there is recent evidence that CYN has teratogenic [25] and reproductive effects [26], and there is preliminary evidence that it may be carcinogenic [20,22,27]. In these circumstances a reasonable additional uncertainty factor of 2 is applicable.

Step 1: TDI = 30 ÷ 200 = 0.15 μg/kg/day

Step 2: Acceptable limit per day = 0.15 μg/kg/day × bodyweight (kg) × 1.0 (allocation factor)

**Table 2:** Acceptable daily limit for cylindrospermopsin with age and bodyweight.

Age group (years)	Average bodyweight (kg)	Acceptable daily limit (μg/day)
≥17	74	11
2–16	38	5.7

Step 3: Obtain high-level consumption data for consumers aged 17 years and above and 2–16 years old—see Table 1.

Step 4: Derive health guideline level (μg/kg) for cylindrospermopsin in whole seafood sample—see Table 3. Acceptable limit (μg/day) ÷ consumption (kg/day) (that is Step 2 ÷ Step 3).

**Table 3:** Health guideline values for cylindrospermopsin toxin in seafood.

Health Guideline Value (μg/kg of whole organism sample)			
Age group (years)	Fish	Prawns	Mussels/Molluscs
≥17	29	29	62
2–16	18	24	39

Preliminary *in vitro* evidence suggests that deoxyCYN has similar potency to CYN, and so this analogue should be included in monitoring programs and toxicity assessments [28,29].

### 3.2. Microcystins

Microcystins have been the most thoroughly investigated cyanobacterial toxin group, and is still the major toxin group under investigation. The majority of human and animal microcystin-related poisonings worldwide have been associated with the presence of the cyanobacterial species *Microcystis aeruginosa and M. flos-aquae*. Microcystins may also be produced by species of the planktonic genera *Anabaena*, *Planktothrix (Oscillatoria)*, *Nostoc*, and *Anabaenopsis*.

The microcystins are a family of cyclic peptide toxins, containing seven peptide-linked amino acids, in which acids in L-configuration occupy two positions in the ring. A range of L-amino acids may take these positions, with consequences for toxicity over a range approaching ten-fold. To standardize guideline values, microcystins toxicity is expressed as toxicity equivalent to microcystin-LR (leucine, arginine) [6].

The most significant recorded human poisoning event due to microcystins occurred in Brazil in 1996 at the Caruaru Dialysis Clinic [30]. Cyanobacterial toxins contaminated the clinic’s water source, so that intravenous exposure to microcystins and cylindrospermopsins during routine renal dialysis treatment led to acute liver failure in 100 patients and resulted in 76 deaths [30]. An Australian study has revealed toxic liver damage (an increase in the activity of the hepatic enzyme–glutamyl-transferase) coincided with a bloom of *M. aeruginosa* in 1981 in a drinking water supply in Armidale NSW [31,32]. At lower doses there is also evidence that microcystin’s effects on cell regulation may increase the growth rate of existing tumours (tumour promotion) [33]. This evidence has been provided by both experimental and epidemiological studies [34] with microcystins and nodularins implicated in tumour promotion in both the liver [35,36] and colon [37]. However, reliable dose-response data for studies recording human exposure to microcystins is lacking, therefore animal studies must be relied upon to derive a TDI.

The only animal study that has met the OECD criteria for subchronic oral toxicity assessment in rodents is that of Fawell *et al.* (1994) [38]. Their 13-week oral gavage study of mice exposure met the criteria for experimental design, duration of exposure and used both sexes of animal. Fawell *et al.* (1994) concluded that the NOAEL for microcystin-LR was 40 μg/kg/day. This is supported by an oral toxicity study carried out in pigs, which resulted in a Lowest Observable Adverse Effect Level (LOAEL) of 100 μg/kg/day of microcystin-LR equivalents [6]. The International Agency for Research on Cancer has recently classified microcystin-LR as a “possible human carcinogen” (Class 2B) [39]. The cited mechanism of action is protein phosphatase inhibition and so the assumption of a threshold dose below that no adverse effect occurs still applies. It is appropriate, therefore, to derive a TDI from the NOAEL.

Thus using 40 μg/kg/day as the NOAEL, the TDI for microcystin-LR (and equivalent toxins) can be calculated:

*TDI (μg/kg/day) = 40 μg/kg/day* *÷ uncertainty factors* (5)

The uncertainty factors are 10 for intraspecies variability, 10 for interspecies variability, and an additional factor of 2 for limitations in data, including evidence of tumour promotion, suspicion of carcinogenesis [39], conflicting data in teratogenesis, and recent evidence of reproductive toxicity.

Step 1: TDI = 40μg/kg/day ÷ 200 = 0.2μg/kg/day.

Step 2: Acceptable limit per day = 0.2μg/kg/day × bodyweight( kg) × 1.0 (allocation factor)

**Table 4:** Acceptable daily limit for microcystins with age and bodyweight.

Age group (years)	Average bodyweight (kg)	Acceptable daily limit (μg/day)
≥17	74	14.8
2–16	38	7.6

Step 3: Apply high level consumption data from Table 1.

Step 4: Derive health guideline level for seafood for microcystin-LR and similar toxins.

**Table 5:** Derived health guideline values for microcystins in seafood.

Health Guideline Value (μg/kg of whole organism sample)			
Age group (years)	Fish	Prawns	Mussels/Molluscs
≥17	39	39	83
2–16	24	32	51

### 3.3. Nodularin

Nodularin is a hepatotoxin produced by *Nodularia spumigena*. *N. spumigena* is primarily regarded as a brackish water species and forms blooms in estuarine lakes in Australia and New Zealand and in the Baltic Sea in Europe [40]. In addition to these saline environments, there have also been frequent blooms in the freshwater lakes of the lower River Murray in South Australia [41]. As a brackish water species *N. spumigena* is the most common toxic cyanobacterial species in the Gippsland Lakes.

Nodularin is structurally very similar to microcystin and has a similar mode of toxicity showing the same hepatotoxic effects through the inhibition of protein phosphatases [42]. Some have suggested it may be more carcinogenic than microcystin [43]. No human poisonings have been recorded as a result of ingestion of *N. spumigena* [31] however it is “at least as hepatotoxic as microcystins for intraperitoneal exposure in experimental animals and, given its identical mode of action, can be regarded as presenting at least the same risk to human health as microcystins if ingested in drinking water” [40]. Due to the structural similarities between microcystins and nodularin and the lack of animal studies looking at the health effects associated with exposure to nodularin it is acceptable that the guideline value for microcystins be applied to nodularin. Several published risk assessments have used a similar approach [5,44]. For calculations, refer to the ‘Microcystins’ section above.

### 3.4. Saxitoxins

There have been no recorded cases of human poisonings as a result of ingestion of saxitoxins produced by cyanobacteria [6]. There are, however, documented cases where saxitoxins arising from dinoflagellates have led to neurotoxic effects as well as death in humans [45]. The established health guideline value for saxitoxins produced by dinoflagellates is 0.8 mg/kg (STX toxicity equivalents) in bivalve mussels (shellfish). This value is used by Food Standards Australia New Zealand (FSANZ Food Standard 1.4.1) and the Victorian Shellfish Quality Assurance Program [46].

Cyanobacteria produce different analogues of saxitoxin as compared to microalgae. In the U.S. *Aphanizomenon* produces saxitoxins, however the only known cyanobacterial producer in Australia is *Anabaena circinalis* [47]. Current evidence suggests that this species produces mainly the less toxic C-toxin analogues, along with lesser amounts of the more toxic analogues commonly found in marine microalgae. However, it is known that acidic or alkaline conditions and heat can chemically convert the C-toxins to the more toxic variants, and that similar bioconversions can occur within shellfish [48]. There is currently no information on the degree of inter-conversion that occurs from these low toxicity variants to the more toxic ones during cooking or digestion in the stomach, although it is known that the more toxic variants are stable at normal cooking temperatures [49].

The health guideline value of 0.8 mg/kg is a long-standing limit that has been used for marine saxitoxins. There has been a long history of success (nearly 50 years) associated with this level, with no evidence of human illnesses from commercially harvested products [45]. Weckell *et al.* attempted to trace the origin of the guideline value and noted that it originated from a U.S. Marine Biotoxins Program [7]. The limit was established in the 1930s ‘based on bioassays measuring toxic activity in mice’, but the exact details of its derivation are uncertain [7]. Despite this, the saxitoxin guideline value has been used by all major regulatory agencies around the world for many years and it does appear to be protective of public health [7].

However, in 2009 the European Food Safety Authority (EFSA) was asked by the European Commission to review the existing ML for saxitoxin. Following a review of published literature EFSA established an ARfD for saxitoxin of 0.5 μg saxitoxin equivalents/kg bw based on human data [49]. Together with an estimate of acute dietary intake for shellfish, EFSA revised the ML down to 75 μg STX equivalents/kg shellfish meat. As the existing saxitoxin ML of 0.8 mg/kg in the Food Standards Code has a long history of effective protection for human health, this substantial downward revision is under further consideration.

## 4. Summary

Our conclusions may be summarized by Table 6.

**Table 6:** Health guideline values for cyanobacterial toxins in seafood (based on consumption by 2–16 year age group).

Toxin	Health guideline value (μg/kg of whole organism sample)		
	Fish	Prawns	Mussels or Molluscs
Cylindrospermopsin and deoxyCYN	18	24	39
Microcystin-LR* or equivalent toxins, incl. Nodularin	24	32	51
Saxitoxins	800	800	800

* The guideline value represents the sum value of all microcystins and nodularin present.
